# Forgotten Poles in the history of neurology: from Flatau to Frey

**DOI:** 10.1055/s-0042-1760086

**Published:** 2022-12-29

**Authors:** Mateusz Gotowiec

**Affiliations:** 1 Medical University of Warsaw, Faculty of Medicine, Warsaw, Poland.

**Keywords:** History, 19th Century, History, 20th Century, History of Medicine, Neurology, Historia del Siglo XIX, Historia del Siglo XX, Historia de la Medicina, Neurología

## Abstract

With the majority of eponyms being removed from disease classification systems, it is even more difficult to remember the neurologists who influenced the development of techniques and understanding of the brain over the last centuries. Determining whether Polish researchers were given similar attention to Western equivalents based on eponymic presence in medical databases is an interesting way to provide an overview of unremembered Polish neurologists. This work aims to recognize the developments of forgotten Polish neurologists, whose work, although important, was not properly appreciated over the centuries.

## Resumen

Con la eliminación de la mayoría de los epónimos de los sistemas de clasificación de enfermedades, es aún más difícil recordar a los neurólogos que influyeron en el desarrollo de técnicas y comprensión del cerebro durante los últimos siglos. Determinar si los investigadores polacos recibieron una atención similar a la de los equivalentes occidentales en función de la presencia de los epónimos en las bases de datos médicos es una forma interesante de proporcionar una visión general de los neurólogos polacos no recordados. Este trabajo tiene como objetivo reconocer los desarrollos de los neurólogos polacos olvidados, cuyo trabajo, aunque importante, no fue debidamente apreciado a lo largo de los siglos.

## DIFFICULTIES OF POLISH NEUROLOGY OVER THE CENTURIES

**Figure 1:**
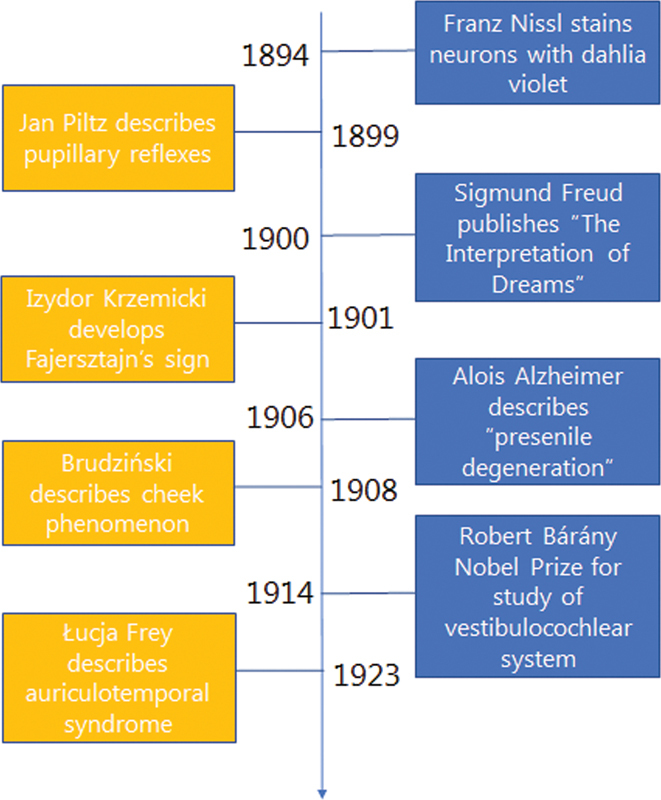
Timeline comparing international and Polish developments in neurology.

The 19 ^th^ century marks the rise of scientific medicine, including the study of the brain. 1 Neurological studies such as the description of *Shaking Palsy* by James Parkinson, in 1817, or the introduction of the term *neuroglia* by Rudolf Virchow, in 1859, are examples of developments that occurred over this important period. 2 Not only Western researchers were responsible for the changes, but nowadays they constitute the most remembered group, specifically when looking at the occurrence of eponyms in the Anglo-Saxon medical setting. During this breakthrough, Poland was partitioned between three superpowers (the German, the Austro-Hungary, and the Russian empires) until 1918, and then occupied during the war period of 1939 to 1945. 3 These difficulties did not favor the expansion of modern medicine; however, they were not able to impede Polish neurologists from developing new treatments ( Figure 1 ). On the other hand, their developments were not followed by international recognition as in many cases annexationists did not allow Polish discoveries to be fully acknowledged. 1 Nowadays, this lack of remembrance is visible when comparing eponymic names commonly found only in Polish medical literature that are not followed by recognition in international databases ( Table 1 ). This article focuses on bringing back the memory of three forgotten Polish neurologists whose stories and lives intertwined throughout the years.

**Table 1:** Comparison between Polish neurological eponyms with those in Anglo-Saxon sources

Polish eponym	Anglo-Saxon name	Description	ICD-11/SNOMED CT	Medical dictionary
Piltz sign	Eye-closure pupil reaction	Constriction of both pupils when trying to close eyelids that are forcibly held apart	✗	**✓**
Fajersztajn-Krzemicki sign	Fajersztajn test	Derivative of Lasègue's sign, occurring as pain in affected limb when testing the healthy one	✓*	✗
Brudziński's sign	Brudzinski's cheek sign	Meningitis determinant based on pressure on check eliciting reflex action that results in twitching of periorbital area and upper lip	✓*	**✓**
Orzechowski sign	Orzechowski syndrome	One of encephalitis signs, nonvoluntary eye oscillations	✗	**✓**
Mackiewicz sign	Femoral nerve stretch test	Characteristic sign of sciatica, when in prone-lying patient flexion at knee joint results in pain of anterior compartment of thigh	✗	✗
Piotrowski sign	Piotrowski sign	Characteristic sign of pyramidal tract lesion, when tapping of tibialis anterior muscle results in plantar flexion of ankle and toes	✗	**✓**
Flatau syndrome	Torsion dystonia	Genetic disease characterized by painful muscle contractions	✗	**✓**
Frey's syndrome	Frey's syndrome	Disorder resulting from damage of parotid gland and auriculotemporal nerve that results in sweating and erythema in response to gustatory stimuli	**✓**	**✓**
Progulski sign	Early meningeal symptom	Lack of smile used to determine early meningitis in children	✗	✗
Herman's sign	Nuchohalux sign	Meningeal sign occurring when passively pulling patient's head toward chest results in dorsiflexion of first toe	✗	✗

Notes: ✓, present; ✗, not present; *, only in SNOMED.

To determine, whether the eponyms, developed by original scientists, 18 are not only used in Polish medical setting but also in dominating Anglo-Saxon medicine, the gathered list was compared with the ICD-11/SNOMED CT and the Free Medical Dictionary. 19

## EDWARD FLATAU – FOUNDER OF POLISH NEUROLOGY

In 1894, 2 years after graduating from the University of Moscow with *cum eximia lauda* distinction, young Edward Flatau published *Atlas des menschlichen Gehirns und des Faserverlaufes* (Atlas of the human brain and the course of the nerve fibers). 4 This extraordinary work, described by Sigmund Freud as “excellent educational material,” was translated into English, Russian, and French shortly after its publication. 5 The atlas became the entry into the world of science for 26-year-old Flatau. Three years later, at the Royal Academy, in Berlin, he showed evidence of the laminar arrangement of spinal pathways stating that “longer spinal tracts have a more eccentric position,” later known as Flatau Law. 5 His respect for patriotism can be seen in his rejection of the position of chair of neurology in Buenos Aires, in 1899, following which he returned to Warsaw in his home region. 5 After an intensive course on neurohistology with Alois Alzheimer and Franz Nissl, in Munich, during which he modified and improved Golgi's method of staining, in 1906, he permanently returned to Warsaw, where he stayed for the next 25 years. 6 During this period, he worked in The Jewish Hospital as head of the department of neurology where, due to his respected position in Russian authority, he developed the most innovative neurological ward in occupied Poland. 7 Under his governance as head of the department of neurobiology of the Warsaw Scientific Society, he established new neurological laboratories to train doctors. 6 He died in 1932, followed 5 months later by Samuel Goldflam, his friend and another important Polish neurologist. 4 Both, through the organization of Polish scientific societies, created space for future scientists to study the brain.

## STANISŁAW PROGULSKI – ONE OF MANY VICTIMS

One of the neurologists who followed Flatau's path through Berlin Charité hospital was 6 years younger Stanisław Progulski, a student of the University of Lwów (currently L'viv, Ukraine) who later developed the Leopolitan School of Thought and profoundly influenced the study of neuroimmunology in children. 8 9 10 Similarly to Flatau, he was of Jewish descent yet also perceived himself as Polish, fighting against Bolshevik forces in L'viv in 1918 as an officer of the Polish Army. 8 After the First World War, he worked as a lecturer at the pediatric clinic where he stayed until 1938. During this period, focusing on the study of bacterial meningitis, he described an early sign of meningitis, that is, lack of smile in children, which later began known as Progulski sign. 8 His thorough commitment to protect the life of newborns led to the promotion of diphtheria vaccination, at first in over 121 orphaned children, and later on a general whole city vaccination action. 8 Unfortunately, his dedication was brutally halted on the 3 ^rd^ of July, 1941, soon after German forces entered L'viv. At midnight, a group of *Einsatzkommando* (secret police) soldiers arrested him, along with other distinguished Leopolitan scientists to “clear the region from particularly dangerous individuals.” He was shot dead the very next day, in what is currently known as Student's Park, not more than two kilometres from his house. 8

## NOT ONLY MEN – THE STORY OF ŁUCJA FREY-GOTTESMAN

Germans, soon after entering L'viv, in 1941, instituted the *Ghetto Lemberg* , dividing the Jewish population into *useful* and *useless* 11 . At first, being a physician, Łucja Frey earned a place in the prime group and worked to ease the suffering of already war-torn people in the *II Ghettopoliklinik* . 12 The last sign of her life comes from April 1942–permission to work No. 144, commonly known as a *green card* . Unfortunately, similarly to Progulski and other Polish Jews, she did not survive *Shoah* . However, her dedication as one of the first Polish female neurologists and academic achievements allowed the memories of her to live on. Her story begins in 1889 in L'viv, where she was born and in 1917 began her medical studies. After 2 years, she moved to Warsaw, completing her education with the highest degree in neurology – her further main interest. Soon after, in 1923 she published her first paper on the auriculotemporal syndrome in the *Polish Medical Journal* followed by the French *Revue Neurologique* . 13 14 Her case report of a 25-year-old-patient with auriculotemporal nerve injury caused by bullet and hyperhidrosis in the facial region when eating proved to be the first such inquisitive study of autonomic innervation of the face. 15 *Gustatory sweating* , caused by erroneous regrowth of parasympathetic fibers into sympathetic receptors, as she proposed, was one of the symptoms of disease, later known as Frey syndrome. 16 17 Her perspicacity as one of the first Polish female neurologists shows a pathway that should still be followed.

**Figure 2:**
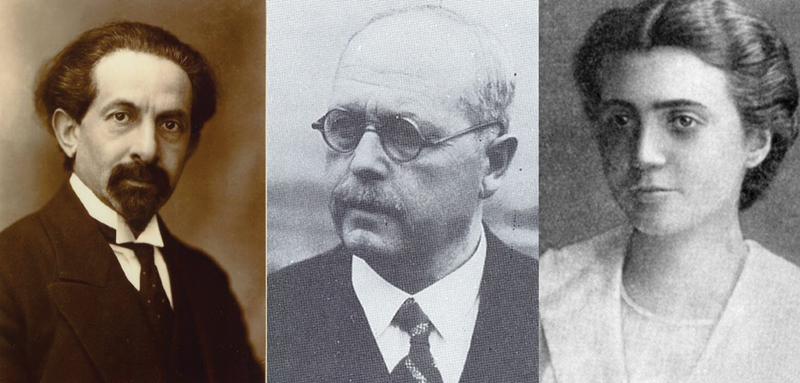
Picture showing the three Polish neurologists described in the text: Edward Flatau, Stanisław Progulski, and Łucja Frey-Gottesman.

In conclusion, the presented personalities ( Figure 2 ) show that modern neurology, although perceived mainly as developed by Western scientists, was not their sole creation. The influence of Polish neurologists and their commitment should never be forgotten and deserves remembrance.
